# Supplementary material for: A Molecular Signature of Circulating MicroRNA Can Predict Osteolytic Bone Disease in Multiple Myeloma
Source: Cancers (Basel). 2021 Jul 31;13(15):3877. doi: 10.3390/cancers13153877 (PMC8345491; doi:10.3390/cancers13153877)
Supplement: Supplementary file 1 [file cancers-13-03877-s001.zip › cancers-1299570-Supplementary.pdf]

Supplementary Materials

A Molecular Signature of Circulating MicroRNA Can Predict Osteolytic Bone Disease in Multiple Myeloma

Aristea-Maria Papanota, Panagiotis Tsiakanikas, Christos K. Kontos, Panagiotis Malandrakis, Christine-Ivy Liacos, Ioannis Ntanasis-Stathopoulos, Nikolaos Kanellias, Maria Gavriatopoulou, Efstathios Kastritis, Margaritis Avgeris, Meletios-Athanasios Dimopoulos, Andreas Scorilas and Evangelos Terpos

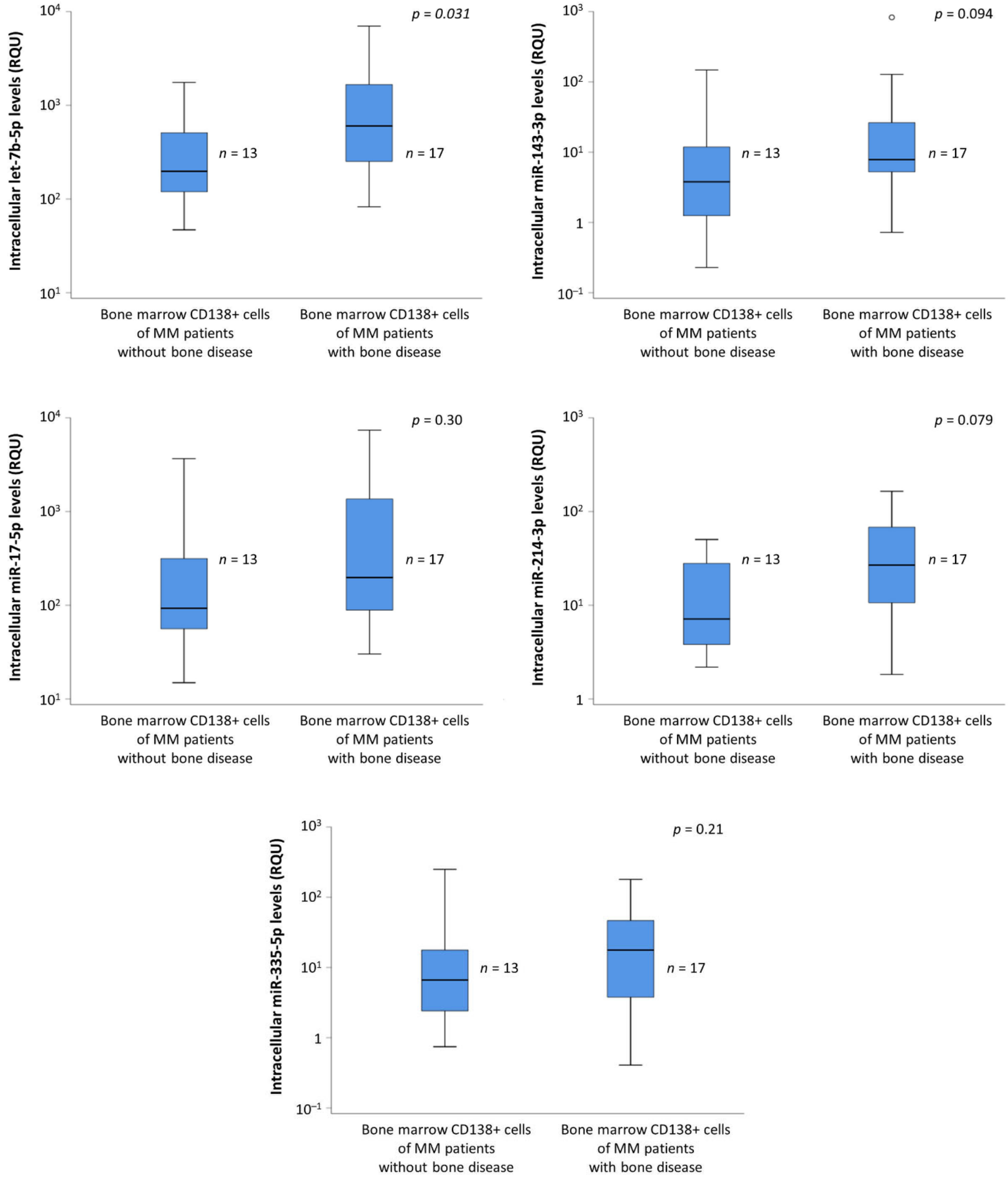

Figure S1. Comparison of intracellular let-7b-5p, miR-143-3p, miR-17-5p, miR-214-3p, and miR-335-5p expression levels in CD138+ plasma cells from BMA samples of MM patients with and without MMBD.

Table S1. Distributions of the levels of circulating let-7b-5p, miR-143-3p, miR-17-5p, miR-214-3p, and miR-335-5p in plasma samples of MM patients and normal controls.

Variable	Mean ± S.E. [1]	Range	Percentiles		
			25th	50th (Median)	75th
let-7b-5p levels (RQU [2])					
in MMBD patients (n=35)	247.52 ± 65.47	5.06 – 2,209.7	50.90	153.24	308.61
in non-MMBD patients (n=27)	101.26 ± 22.15	11.31 – 491.02	36.75	58.07	99.71
in normal controls (n=10)	93.66 ± 20.45	11.71 – 227.49	57.67	74.53	121.29
miR-143-3p levels (RQU [2])					
in MMBD patients (n=32)	15.65 ± 5.88	0.010 – 157.55	0.23	1.62	12.29
in non-MMBD patients (n=24)	1.39 ± 0.52	0.010 – 11.96	0.10	0.64	1.31
in normal controls (n=10)	1.28 ± 0.54	0.020 – 5.66	0.12	0.94	1.54
miR-17-5p levels (RQU [2])					
in MMBD patients (n=33)	59.49 ± 34.80	0.010 – 1,159.8	2.54	8.63	42.43
in non-MMBD patients (n=26)	10.72 ± 3.08	0.030 – 63.10	1.05	2.49	15.79
in normal controls (n=10)	10.14 ± 3.47	0.32 – 30.48	1.06	5.28	19.28
miR-214-3p levels (RQU [2])					
in MMBD patients (n=29)	5.13 ± 4.05	0.020 – 118.57	0.43	0.78	2.10
in non-MMBD patients (n=22)	0.46 ± 0.11	0.020 – 2.20	0.10	0.24	0.53
in normal controls (n=10)	0.59 ± 0.20	0.11 – 2.10	0.16	0.34	0.94
miR-335-5p levels (RQU [2])					
in MMBD patients (n=31)	22.28 ± 14.46	0.17 – 451.83	0.81	2.58	15.13
in non-MMBD patients (n=24)	1.58 ± 0.29	0.040 – 5.70	0.63	1.18	2.21
in normal controls (n=10)	1.83 ± 0.42	0.11 – 4.79	0.68	1.86	2.31

[1] Standard error. [2] Relative quantification units.
